# Supplementary material for: Manipulating type-I and type-II Dirac polaritons in cavity-embedded honeycomb metasurfaces
Source: Nat Commun. 2018 Jun 6;9:2194. doi: 10.1038/s41467-018-03982-7 (PMC5989260; doi:10.1038/s41467-018-03982-7)
Supplement: Supplementary file 1 — Supplementary Information [file 41467_2018_3982_MOESM1_ESM.pdf]

Supplementary Information:

Manipulating Type-I and Type-II Dirac Polaritons in Cavity-Embedded Honeycomb Metasurfaces

Mann et al.

SUPPLEMENTARY NOTE 1

Including all Coulomb interactions beyond the nearest-neighbor approximation leads to the full matter Hamiltonian within the dipole approximation

$$\begin{aligned} H_{\text{mat}} &= \hbar\omega_0 \sum_{\mathbf{q}} \left(a_{\mathbf{q}}^\dagger a_{\mathbf{q}} + b_{\mathbf{q}}^\dagger b_{\mathbf{q}}\right) + \hbar\Omega \sum_{\mathbf{q}} \left[f_{\mathbf{q}}^{\text{AB}} b_{\mathbf{q}}^\dagger \left(a_{\mathbf{q}} + a_{-\mathbf{q}}^\dagger\right) + \text{H.c.}\right] \\ &+ \frac{\hbar\Omega}{2} \sum_{\mathbf{q}} \left[f_{\mathbf{q}}^{\text{AA}} a_{\mathbf{q}}^\dagger \left(a_{\mathbf{q}} + a_{-\mathbf{q}}^\dagger\right) + \text{H.c.}\right] + \frac{\hbar\Omega}{2} \sum_{\mathbf{q}} \left[f_{\mathbf{q}}^{\text{BB}} b_{\mathbf{q}}^\dagger \left(b_{\mathbf{q}} + b_{-\mathbf{q}}^\dagger\right) + \text{H.c.}\right], \end{aligned} \tag{1}$$

where

$$f_{\mathbf{q}}^{\text{AA}} = f_{\mathbf{q}}^{\text{BB}} = -\mathcal{S} + \sum_{\mathbf{R}\neq 0} \left(\frac{a}{|\mathbf{R}|}\right)^3 \left(1 - \mathcal{I}_{\mathbf{R}}^{\text{AA}}\right) \mathrm{e}^{\mathrm{i}\mathbf{q}\cdot\mathbf{R}} \tag{2}$$

and

$$f_{\mathbf{q}}^{\text{AB}} = \sum_{\mathbf{R}} \left(\frac{a}{|\mathbf{R}+\mathbf{e}_1|}\right)^3 \left(1 - \mathcal{I}_{\mathbf{R}}^{\text{AB}}\right) \mathrm{e}^{\mathrm{i}\mathbf{q}\cdot(\mathbf{R}+\mathbf{e}_1)}. \tag{3}$$

Here, the parameters

$$\mathcal{I}_{\mathbf{R}}^{\text{AA}} = 2\sum_{l=1}^{\infty} \frac{2\left(\frac{lL}{|\mathbf{R}|}\right)^2 - 1}{\left[1 + \left(\frac{lL}{|\mathbf{R}|}\right)^2\right]^{\frac{5}{2}}}, \qquad \mathcal{I}_{\mathbf{R}}^{\text{AB}} = 2\sum_{l=1}^{\infty} \frac{2\left(\frac{lL}{|\mathbf{R}+\mathbf{e}_1|}\right)^2 - 1}{\left[1 + \left(\frac{lL}{|\mathbf{R}+\mathbf{e}_1|}\right)^2\right]^{\frac{5}{2}}} \tag{4}$$

encode the renomalizations due to the interaction with cavity-induced image dipoles of neighboring meta-atoms, and $\mathcal{S}$, given by equation (16) in the Methods, encodes renormalizations due to interactions with their self-images. We can recast the matter Hamiltonian into matrix form as $H_{\text{mat}} = \frac{1}{2}\sum_{\mathbf{q}} \Psi_{\mathbf{q}}^\dagger \mathscr{H}_{\mathbf{q}}^{\text{mat}} \Psi_{\mathbf{q}}$, where $\Psi_{\mathbf{q}}^\dagger = (\psi_{\mathbf{q}}^\dagger, \psi_{-\mathbf{q}}^{\text{T}})$ and $\psi_{\mathbf{q}}^\dagger = (a_{\mathbf{q}}^\dagger, b_{\mathbf{q}}^\dagger)$. Here, the Hermitian 4×4 matrix $\mathscr{H}_{\mathbf{q}}^{\text{mat}}$ can be written in block form as

$$\mathscr{H}_{\mathbf{q}}^{\text{mat}} = \begin{pmatrix} \mathcal{H}_{\mathbf{q}}^{\text{mat}} & \mathcal{H}_{\mathbf{q}}^{\text{mat}} - \hbar\omega_0 \mathbb{1}_2 \\ \mathcal{H}_{\mathbf{q}}^{\text{mat}} - \hbar\omega_0 \mathbb{1}_2 & \mathcal{H}_{\mathbf{q}}^{\text{mat}} \end{pmatrix}, \tag{5}$$

with

$$\mathcal{H}_{\mathbf{q}}^{\text{mat}} = \hbar \begin{pmatrix} \omega_0 + \Omega\,\text{Re}\left(f_{\mathbf{q}}^{\text{AA}}\right) & \Omega\left(f_{\mathbf{q}}^{\text{AB}}\right)^* \\ \Omega f_{\mathbf{q}}^{\text{AB}} & \omega_0 + \Omega\,\text{Re}\left(f_{\mathbf{q}}^{\text{BB}}\right) \end{pmatrix}. \tag{6}$$

We diagonalize H_{mat} via a Bogoliubov transformation $\Psi_{\mathbf{q}} = T_{\mathbf{q}} X_{\mathbf{q}}$, where $X_{\mathbf{q}}^\dagger = (\chi_{\mathbf{q}}^\dagger, \chi_{-\mathbf{q}}^{\text{T}})$ and $\chi_{\mathbf{q}}^\dagger = (\beta_{\mathbf{q}+}^\dagger, \beta_{\mathbf{q}-}^\dagger)$, with $T_{\mathbf{q}}$ being a paraunitary matrix (see Methods). The bosonic operators $\beta_{\mathbf{q}\tau}^\dagger = (\cosh\vartheta_{\mathbf{q}\tau}\psi_{\mathbf{q}}^\dagger - \sinh\vartheta_{\mathbf{q}\tau}\psi_{-\mathbf{q}}^{\text{T}})|\psi_{\mathbf{q}\tau}\rangle$ diagonalize the matter Hamiltonian as

$$H_{\text{mat}} = \sum_{\tau=\pm}\sum_{\mathbf{q}} \hbar\omega_{\mathbf{q}\tau}^{\text{mat}} \beta_{\mathbf{q}\tau}^\dagger \beta_{\mathbf{q}\tau}, \tag{7}$$

with quasistatic dispersion

$$\omega_{\mathbf{q}\tau}^{\text{mat}} = \sqrt{\omega_0 + 2\Omega\,\text{Re}\left(f_{\mathbf{q}}^{\text{AA}}\right) + 2\tau\Omega\left|f_{\mathbf{q}}^{\text{AB}}\right|}. \tag{8}$$

and spinors $|\psi_{\mathbf{q}\tau}\rangle = (1, \tau\mathrm{e}^{\mathrm{i}\varphi_{\mathbf{q}}})^{\text{T}}/\sqrt{2}$, where $\varphi_{\mathbf{q}} = \arg(f_{\mathbf{q}}^{\text{AB}})$. The coefficients $\cosh\vartheta_{\mathbf{q}\tau} = (\omega_0 + \omega_{\mathbf{q}\tau}^{\text{mat}})/2\sqrt{\omega_0\omega_{\mathbf{q}\tau}^{\text{mat}}}$ and $\sinh\vartheta_{\mathbf{q}\tau} = (\omega_0 - \omega_{\mathbf{q}\tau}^{\text{mat}})/2\sqrt{\omega_0\omega_{\mathbf{q}\tau}^{\text{mat}}}$ describe the contributions of the resonant and non-resonant terms, respectively. Neglecting non-resonant terms we obtain $H_{\text{mat}} = \sum_{\mathbf{q}} \psi_{\mathbf{q}}^\dagger \mathcal{H}_{\mathbf{q}}^{\text{mat}} \psi_{\mathbf{q}}$ which can be diagonalized as $H_{\text{mat}} = \sum_{\tau=\pm}\sum_{\mathbf{q}} \hbar\omega_{\mathbf{q}\tau}^{\text{mat}} \beta_{\mathbf{q}\tau}^\dagger \beta_{\mathbf{q}\tau}$, where the operators $\beta_{\mathbf{q}\tau}^\dagger = \psi_{\mathbf{q}}^\dagger |\psi_{\mathbf{q}\tau}\rangle$ and the corresponding dispersion $\omega_{\mathbf{q}\tau}^{\text{mat}} = \omega_0 + \Omega\,\text{Re}\left(f_{\mathbf{q}}^{\text{AA}}\right) + \tau\Omega\left|f_{\mathbf{q}}^{\text{AB}}\right|$ take a simpler form.

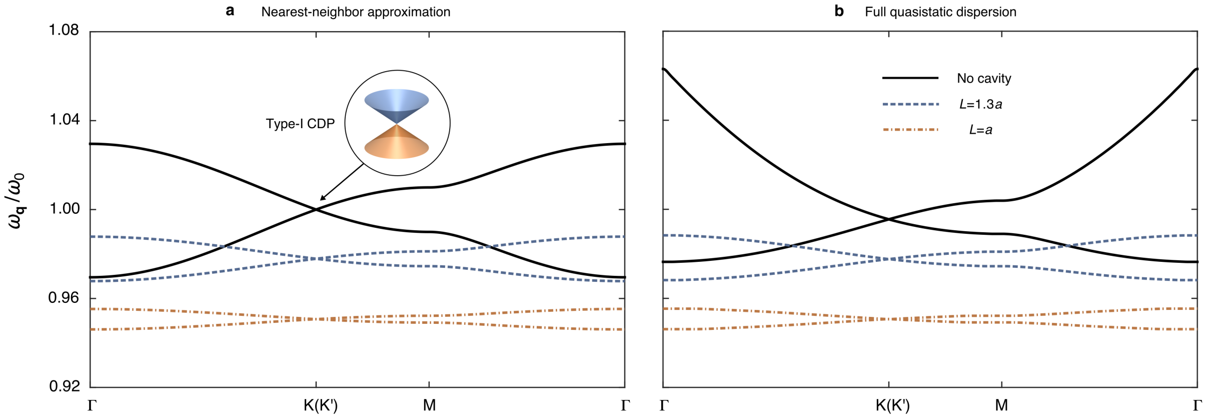

Supplementary Figure 1. **Quasistatic dispersion beyond the nearest-neighbor approximation.** **a**, Quasistatic dispersion within the nearest-neighbor approximation for cavity heights of $L = 1.3a$ (blue dashed line) and $L = a$ (orange dot-dashed line), where a is the nearest-neighbor separation. For comparison we have also plotted the quasistatic dispersion without a cavity (black solid line). One observes that the effect of the image dipoles is to renormalize the resonant frequency and the Coulomb interaction strength, resulting in the quasistatic dispersion shifting down in frequency and reducing in bandwidth. Panel **b** is the same as **a** but with the full quasistatic dispersion [see Supplementary Equation (8)] that includes Coulomb interactions beyond nearest-neighbors until convergence is reached. Long-range Coulomb interactions introduce an asymmetry between the upper and lower quasistatic bands as shown in **b**, but the type-I CDPs remain robust at the K and K' points. As the cavity height is reduced, the image dipoles quench long-range Coulomb interactions, which become negligible for cavity heights on the order of the nearest-neighbor separation. All plots obtained with $\Omega = 0.01\omega_0$.

In Supplementary Figure 1a we plot the quasistatic dispersion within the nearest-neighbor approximation for different cavity heights and compare it to the full quasistatic dispersion in Supplementary Figure 1b, which is given by Supplementary Equation (8). Indeed, due to the honeycomb symmetry, one observes that the type-I CDPs remain pinned at the K and K' points. To prove this robustness against long-range Coulomb interactions, one must show that the function $f_{\mathbf{q}}^{\mathrm{AB}}$, given by Supplementary Equation (3), vanishes at the corners of the Brillouin zone, i.e., one must show that the phase factors associated with the interactions with dipoles residing at the same separation distance and different sublattices sum to zero. To this aim, we define separation vectors $\mathbf{A}_{l_1 l_2} = l_1\mathbf{a}_1 + l_2\mathbf{a}_2 + \mathbf{e}_3$ that connect meta-atoms residing on different sublattices, with primitive lattice vectors $\mathbf{a}_1 = a(\sqrt{3}, 0)$ and $\mathbf{a}_2 = a(-\frac{\sqrt{3}}{2}, \frac{3}{2})$, and integers l_1 and l_2. We can then find six separation vectors with the same magnitude by exploiting the three-fold rotational symmetry $\mathbf{A}_{l_1 l_2}^{j+} = O_j \mathbf{A}_{l_1 l_2}$, where the operator O_j rotates a vector by the angle $2\pi j/3$ ($j = 0, 1, 2$), and the mirror symmetry about the y-axis $\mathbf{A}_{l_1 l_2}^{j-} = M_x \mathbf{A}_{l_1 l_2}^{j+}$, where the operator M_x inverts the sign of the x component. Explicitly, the six separation vectors (not necessarily all different) read

$$\mathbf{A}_{l_1 l_2}^{0\pm} = \pm a \left[\frac{\sqrt{3}}{2}(2l_1 - l_2 - 1)\right]\hat{\mathbf{x}} + a\left[\frac{1}{2}(3l_2 + 1)\right]\hat{\mathbf{y}} \tag{9}$$

$$\mathbf{A}_{l_1 l_2}^{1\pm} = \pm a \left[\frac{\sqrt{3}}{2}(-l_1 - l_2)\right]\hat{\mathbf{x}} + a\left[\frac{1}{2}(3l_1 - 3l_2 - 1)\right]\hat{\mathbf{y}} \tag{10}$$

$$\mathbf{A}_{l_1 l_2}^{2\pm} = \pm a \left[\frac{\sqrt{3}}{2}(-l_1 + 2l_2 + 1)\right]\hat{\mathbf{x}} + a\left[\frac{1}{2}(-3l_1 + 1)\right]\hat{\mathbf{y}} \tag{11}$$

and evaluating Supplementary Equation (3) at $\mathbf{K}$ one indeed finds $f_{\mathbf{K}}^{\mathrm{AB}} = 0$.

Without a cavity, the $f_{\mathbf{q}}^{\mathrm{AA}}$ term, given by Supplementary Equation (2), introduces strong asymmetry between the upper and lower quasistatic bands. Since the lower band corresponds to a dark ($\uparrow\downarrow$) configuration of the two sublattices, the dispersion converges rapidly with increasing number of interactions taken into account as long-range Coulomb interactions quickly average to zero. The most prominent change is seen in the upper band where the group velocity increases towards the Γ point, which is in contrast to the dispersion within the nearest-neighbor approximation. This is because the upper quasistatic band corresponds to a bright ($\uparrow\uparrow$) configuration of the two sublattices and therefore long-range Coulomb interactions do not average to zero, especially near the Γ point. However, as the cavity height is reduced, the image dipoles quench long-range Coulomb interactions, which eventually become negligible for cavity heights on the order of the nearest-neighbor separation.

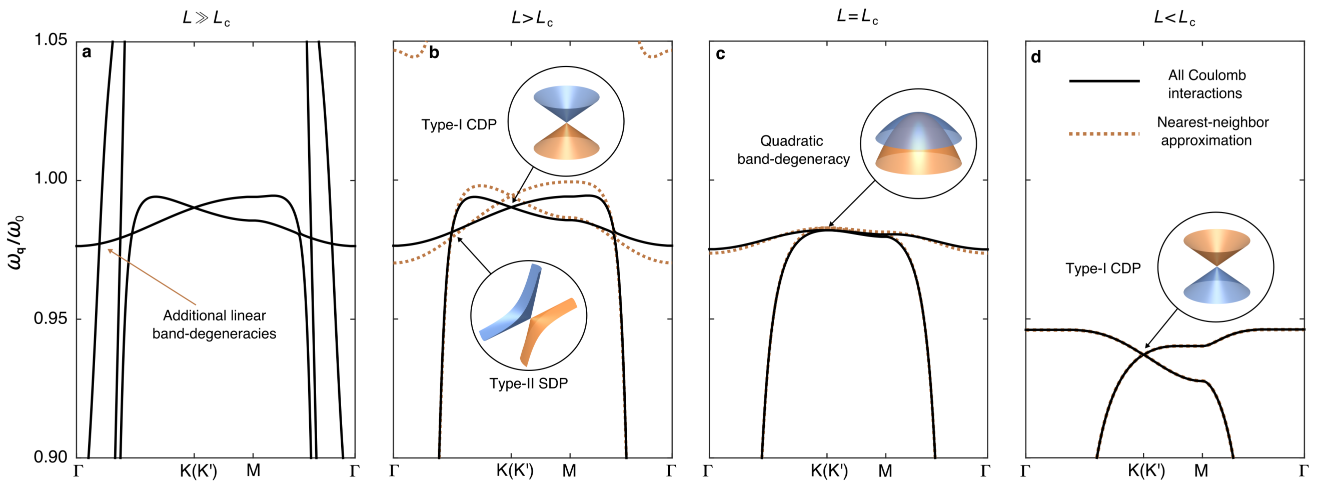

Supplementary Figure 2. **Evolution of the polariton dispersion beyond the nearest-neighbor approximation.** Full polariton dispersion that includes long-range Coulomb interactions (solid black lines) compared to the polariton dispersion obtained within the nearest-neighbor approximation (orange dashed lines) for **a** very large ($L = 15a$), **b** sub-critical ($L = 5a$), **c** critical ($L = 1.75a$), and **d** super-critical ($L = a$) cavity heights, respectively, where a is the nearest-neighbor separation. Higher-order cavity modes can be observed for very large cavities as seen in **a**. Along the $\Gamma - \mathrm{K}(\mathrm{K}')$ directions the band crossings between the photon dispersion and the lower quasistatic band are protected, which gives rise to many additional linear-band degeneracies within the light-cone. As the cavity height is decreased, these additional Dirac points are driven towards the Γ point (opposite direction to the SDPs emerging from the TEM mode) where they annihilate one another. Comparing panels **a** and **b**, one can see that the dispersion near the CDPs does not noticeably change, and qualitative changes outside the light-cone only occur for small cavity heights where the higher-order cavity modes become detuned from the dipole resonances (see panels **b**-**d**). For small cavity heights, the nearest-neighbor approximation becomes increasingly accurate and indistinguishable from the full polariton dispersion (see **d** for example). Plots obtained with parameters $\omega^{\mathrm{ph}}_{\mathbf{K}0} = 2.5\omega_0$ and $\Omega = 0.01\omega_0$.

The polariton dispersion plots presented in the main text (Fig. 2c-e) are within the nearest-neighbor approximation. In Supplementary Figure 2a-d we present the polariton dispersion including all Coulomb interactions beyond nearest-neighbors until convergence is reached, i.e., using Supplementary Equation (6) instead of equation (28) in the polariton Hamiltonian (see Methods). As expected from the quasistatic dispersion plots in Supplementary Figure 1b, long-range Coulomb interactions do not qualitatively affect the physics near the K and K' points. In fact, for small cavity heights, the nearest-neighbor approximation becomes increasingly accurate and indistinguishable from the full polariton dispersion (see Supplementary Figure 2d for example).

As described in the main text, six inequivalent type-II SDPs emerge in the polariton spectrum due to the protected crossing with the TEM mode along the $\Gamma - \mathrm{K}(\mathrm{K}')$ directions. Protected crossings with higher-order cavity modes ($m \neq 0$) result in a large number of additional type-II Dirac points being generated within the light-cone for large cavity heights (see Supplementary Figure 2a). However, as the cavity height is decreased, these additional Dirac points migrate towards the Γ point (opposite direction to the SDPs emerging from the TEM mode), where they eventually merge with and annihilate one another. Comparing Supplementary Figure 2a and Supplementary Figure 2b, one observes that the polariton dispersion near the CDPs does not noticeably change, and qualitative changes outside the light-cone only occur at small cavity heights where the higher-order cavity modes become detuned from the dipole resonances.

SUPPLEMENTARY NOTE 2

In the Methods section we performed a unitary Schrieffer-Wolff transformation on the polariton Hamiltonian and obtained a two-band Hamiltonian in the matter sublattice space given by equation (36). In this section, we seek an effective Hamiltonian describing the polaritons near the corners of the Brillouin zone by expanding the two-band Bloch Hamiltonian (37) near the K point (see Methods). To quadratic order in $\mathbf{k} = \mathbf{q} - \mathbf{K}$, the diagonal matrix elements expand as $W_{\mathbf{q}} \simeq W^{(0)}_{\mathrm{K},\mathbf{k}} + W^{(1)}_{\mathrm{K},\mathbf{k}} + W^{(2)}_{\mathrm{K},\mathbf{k}}$ and the off-diagonal elements as $F_{\mathbf{q}} \simeq F^{(0)}_{\mathrm{K},\mathbf{k}} + F^{(1)}_{\mathrm{K},\mathbf{k}} + F^{(2)}_{\mathrm{K},\mathbf{k}}$, where the superscript index denotes the corresponding order in $\mathbf{k}$. To determine the real and imaginary components in the expansion, we must sum the phase contributions from the degenerate photons at the K point. To this aim, we define reciprocal lattice vectors $\mathbf{G}_n = n_1\mathbf{b}_1 + n_2\mathbf{b}_2$ with primitive vectors $\mathbf{b}_1 =$

$\frac{2\pi}{3a}(\sqrt{3},-1)$ and $\mathbf{b}_2=\frac{4\pi}{3a}(0,1)$. In analogy with the analysis in Supplementary Note 1, we can find the reciprocal lattice vectors associated with six degenerate photonic modes at K by exploiting the three-fold rotational symmetry of the reciprocal lattice $\mathbf{G}_n^{j+}=\mathbf{K}-O_j(\mathbf{K}-\mathbf{G}_n)$, where the operator O_j rotates a vector by the angle $2\pi j/3$ ($j=0,1,2$), and the mirror symmetry about the $k_y=0$ line $\mathbf{G}_n^{j-}=M_y\mathbf{G}_n^{j+}$, where the operator M_y inverts the sign of the y-component. Explicitly, the six reciprocal lattice vectors (not necessarily all different) read

$$\mathbf{G}_n^{0\mu}=\mathbf{K}+\left[\frac{2\pi}{3\sqrt{3}a}(3n_1-2)\right]\hat{\mathbf{x}}+\mu\left[\frac{2\pi}{3a}(2n_2-n_1)\right]\hat{\mathbf{y}}, \tag{12}$$

$$\mathbf{G}_n^{1\mu}=\mathbf{K}+\left[\frac{2\pi}{3\sqrt{3}a}(1-3n_2)\right]\hat{\mathbf{x}}+\mu\left[\frac{2\pi}{3a}(2n_1-n_2-1)\right]\hat{\mathbf{y}}, \tag{13}$$

$$\mathbf{G}_n^{2\mu}=\mathbf{K}+\left[\frac{2\pi}{3\sqrt{3}a}(1+3n_2-3n_1)\right]\hat{\mathbf{x}}+\mu\left[\frac{2\pi}{3a}(1-n_1-n_2)\right]\hat{\mathbf{y}}, \tag{14}$$

with $\mu=\pm1$. Exploiting these symmetries one can rewrite the summations over photonic indices as

$$\sum_{mn}\Delta_{\mathbf{K}mn}^{(v)}\cdots\rightarrow\frac{1}{6}\sum_{mn}\Delta_{\mathbf{K}mn}^{(v)}\sum_{j=0}^{2}\sum_{\mu=\pm}\cdots \tag{15}$$

where the factor of $1/6$ is introduced to avoid over counting. First, the constant (zeroth order) term in the expansion of $W_{\mathbf{q}}$ is

$$W_{\mathrm{K},\mathbf{k}}^{(0)}=\omega_0-\Omega\mathcal{S}-\frac{\Omega}{6}\sum_{mn}\Delta_{\mathbf{K}mn}^{(0)}\sum_{j=0}^{2}\sum_{\mu=\pm}1=\bar{\omega}_0. \tag{16}$$

The linear (first order) term vanishes as

$$W_{\mathrm{K},\mathbf{k}}^{(1)}=\frac{\Omega a^2}{6}\sum_{mn}\Delta_{\mathbf{K}mn}^{(1)}\sum_{j=0}^{2}\sum_{\mu=\pm}\left[(\mathbf{K}-\mathbf{G}_n^{j\mu})_x k_x+(\mathbf{K}-\mathbf{G}_n^{j\mu})_y k_y\right]=0, \tag{17}$$

which illustrates that the light-matter interaction does not introduce anisotropic warping of the Dirac cone to first order. Finally, the quadratic (second order) term is

$$\begin{aligned}W_{\mathrm{K},\mathbf{k}}^{(2)}=&-\frac{\Omega a^4}{6}\sum_{mn}\Delta_{\mathbf{K}mn}^{(2)}\sum_{j=0}^{2}\sum_{\mu=\pm}(\mathbf{K}-\mathbf{G}_n^{j\mu})_x(\mathbf{K}-\mathbf{G}_n^{j\mu})_y k_x k_y\\&-\frac{\Omega a^4}{12}\sum_{mn}\Delta_{\mathbf{K}mn}^{(2)}\sum_{j=0}^{2}\sum_{\mu=\pm}\left[(\mathbf{K}-\mathbf{G}_n^{j\mu})_x^2k_x^2+(\mathbf{K}-\mathbf{G}_n^{j\mu})_y^2k_y^2\right]\\&+\frac{\Omega a^2}{12}\sum_{mn}\Delta_{\mathbf{K}mn}^{(1)}\sum_{j=0}^{2}\sum_{\mu=\pm}(k_x^2+k_y^2)\\=&-D(k_x^2+k_y^2),\end{aligned} \tag{18}$$

The constant (zeroth order) term in the expansion of $F_{\mathbf{q}}$ vanishes at the high-symmetry points as

$$F_{\mathrm{K},\mathbf{k}}^{(0)}=-\frac{\Omega}{6}\sum_{mn}\Delta_{\mathbf{K}mn}^{(0)}\sum_{j=0}^{2}\sum_{\mu=\pm}\phi_{nj\mu}^2=0, \tag{19}$$

where $\phi_{nj\mu}=\exp\left(\mathrm{i}a\mathbf{G}_n^{j\mu}\cdot\hat{\mathbf{y}}\right)$. This shows that the CDPs remain pinned at the K and K′ points and are completely robust against the coupling to the surrounding photonic environment. The linear (first order) term reads

$$\begin{aligned}F_{\mathrm{K},\mathbf{k}}^{(1)}=&-\frac{3\Omega a}{2}(1-\mathcal{I})\left(k_x+\mathrm{i}k_y\right)+\frac{\Omega a^2}{6}\sum_{mn}\Delta_{\mathbf{K}mn}^{(1)}\sum_{j=0}^{2}\sum_{\mu=\pm}\phi_{nj\mu}^2\left[(\mathbf{K}-\mathbf{G}_n^{j\mu})_x k_x+(\mathbf{K}-\mathbf{G}_n^{j\mu})_y k_y\right]\\=&-\bar{v}(k_x+\mathrm{i}k_y),\end{aligned} \tag{20}$$

Finally, the quadratic (second order) term is given by

$$\begin{aligned}F^{(2)}_{\mathrm{K},\mathbf{k}} =& \frac{3\Omega a^2}{8}(1-\mathcal{I})\left(k_x - \mathrm{i}k_y\right)^2 + \frac{\Omega a^2}{12}\sum_{mn}\Delta^{(1)}_{\mathbf{K}mn}\sum_{j=0}^{2}\sum_{\mu=\pm}\phi^2_{nj\mu}(k_x^2+k_y^2)\\ &- \frac{\Omega a^4}{12}\sum_{mn}\Delta^{(2)}_{\mathbf{K}mn}\sum_{j=0}^{2}\sum_{\mu=\pm}\phi^2_{nj\mu}\left[(\mathbf{K}-\mathbf{G}^{j\mu}_n)^2_x k_x^2 + (\mathbf{K}-\mathbf{G}^{j\mu}_n)^2_y k_y^2\right]\\ &- \frac{\Omega a^4}{6}\sum_{mn}\Delta^{(2)}_{\mathbf{K}mn}\sum_{j=0}^{2}\sum_{\mu=\pm}\phi^2_{nj\mu}(\mathbf{K}-\mathbf{G}^{j\mu}_n)_x(\mathbf{K}-\mathbf{G}^{j\mu}_n)_y k_x k_y\\ =& \,\bar{t}\left(k_x - \mathrm{i}k_y\right)^2 .\end{aligned} \tag{21}$$

We can now identify the effective Hamiltonian $\bar{H}^{\mathrm{eff}}_{\mathrm{K}} = \sum_{\mathbf{k}}\psi^{\dagger}_{\mathbf{k}}\bar{\mathcal{H}}^{\mathrm{eff}}_{\mathrm{K},\mathbf{k}}\psi_{\mathbf{k}}$ in the matter sublattice space, where the effective Bloch Hamiltonian $\bar{\mathcal{H}}^{\mathrm{eff}}_{\mathrm{K},\mathbf{k}}$ is given by equation (9) in the main text.

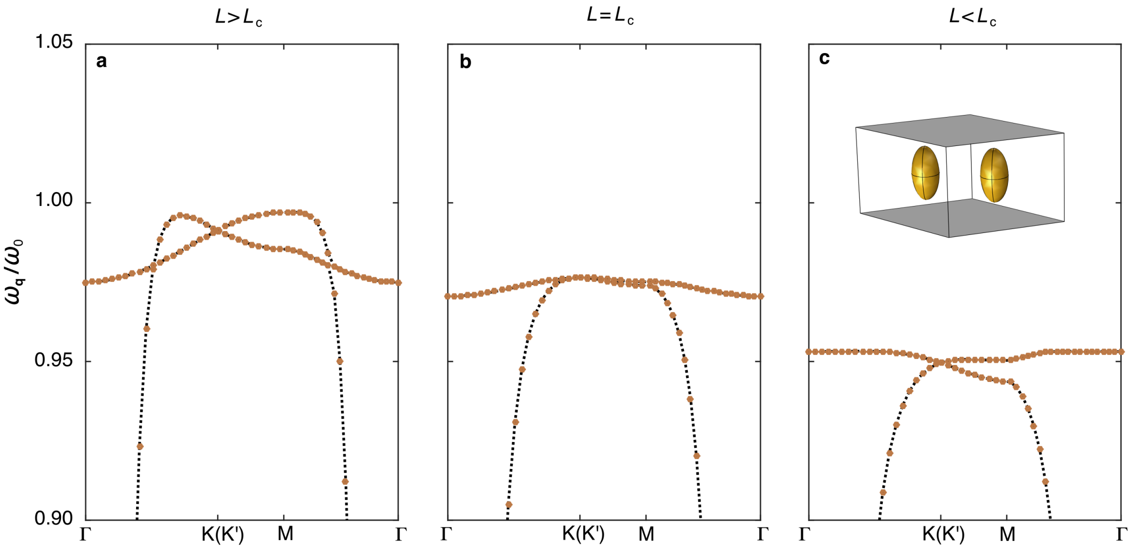

Supplementary Figure 3. **Numerical simulation of the polariton dispersion for a cavity-embedded honeycomb array of plasmonic nanorods**. We consider ellipsoidal nanorods (prolate spheroids) with semiminor axis and semimajor axis of lengths $10\,\mathrm{nm}$ and $20\,\mathrm{nm}$, respectively, and model them using the simple Drude model permittivity with plasma frequency $\omega_{\mathrm{p}} = 1.32\times10^{16}\,\mathrm{rad\,s^{-1}}$ and nonradiative damping frequency $\gamma = 6.9\times10^{13}\,\mathrm{s^{-1}}$. The plasmonic nanorods are arranged in a honeycomb array with nearest-neighbor separation $a = 45\,\mathrm{nm}$. We apply perfect electric conductor boundary conditions for the cavity walls and periodic boundary conditions surrounding the unit cell (see inset on panel **c**). **a**-**c**, Numerical simulations of the polariton dispersion for the lowest two bands, calculated using the eigenmode solver in COMSOL Multiphysics, for cavity heights of **a** $L = 3a$, **b** $L = 1.7a$, and **c** $L = 1.3a$, respectively. The orange dots represent numerical solutions and the dashed line is a guide for the eye. The frequencies are normalized with respect to $\omega_0 = 5.3\times10^{15}\,\mathrm{rad\,s^{-1}}$.

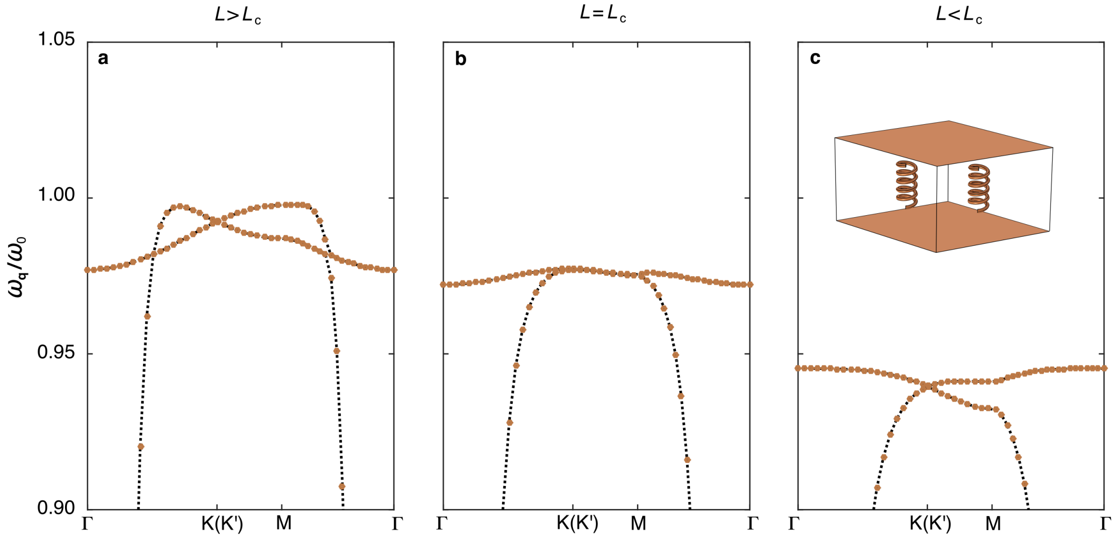

Supplementary Figure 4. **Numerical simulation of the polariton dispersion for a cavity-embedded honeycomb array of microwave helical resonators**. We consider microwave helical resonators with minor radius $2\,\mathrm{mm}$, major radius $6\,\mathrm{mm}$, pitch $6\,\mathrm{mm}$, and 5 turns (see inset on panel **c**). We model the helices and cavity walls as perfect electric conductors, which is a good approximation at microwave frequencies, and we apply periodic boundary conditions surrounding the unit cell. The helices are arranged in a honeycomb array with nearest-neighbor separation $a = 45\,\mathrm{mm}$. **a**-**c**, Numerical simulations of the polariton dispersion for the lowest two bands, calculated using the eigenmode solver in COMSOL Multiphysics, for cavity heights of **a** $L = 3.33a$, **b** $L = 1.56a$, and **c** $L = 1.11a$, respectively. The orange dots represent numerical solutions and the dashed line is a guide for the eye. The frequencies are normalized with respect to $\omega_0 = 6.2 \times 10^9\,\mathrm{rad\,s^{-1}}$.
